# Protocol for obtaining doubled haploids in isolated microspore culture *in vitro* for poorly responsive genotypes of brassicaceae family

**DOI:** 10.1093/biomethods/bpae091

**Published:** 2024-12-03

**Authors:** Elena V Kozar, Elena A Domblides

**Affiliations:** Federal State Budgetary Scientific Institution Federal Scientific Vegetable Center (FSBSI FSVC), Selektsionnaya St, 14, VNIISSOK, Odintsovo Reg., 143072 Moscow, Russia; Federal State Budgetary Scientific Institution Federal Scientific Vegetable Center (FSBSI FSVC), Selektsionnaya St, 14, VNIISSOK, Odintsovo Reg., 143072 Moscow, Russia

**Keywords:** androgenesis, doubled haploid, embryoids; insulation, mustard, rapeseed, radish, red cabbage, technology improvement, white cabbage

## Abstract

In this protocol for obtaining doubled haploids plants (DH), we propose a new method for microspore isolation. This method is useful for genotypes of the Brassicaceae family with low responsiveness to DH technology. For such crops, it allows increasing the embryo yield several times and sometimes obtaining embryos for the first time. This method of microspore isolation reduces the mechanical impact on the bud tissue, which minimizes somatic cell destruction and reduces to get it into the preparation through the filter, thus increasing its purity. The new isolation method also increases the relative concentration of embryogenic microspores in the preparation. This is possible because the anther tissues are not destroyed during the isolation process. Therefore, the anther retains its structure and microspores of early and late stages are trapped by the anther tissue, thus the anther acts as a sieve. Late stages are trapped because of their larger size, while early stages are trapped because they are even more tightly bound to the anther tissue. Together, these factors increase the efficiency of the technology for DH production *in vitro* microspore culture. This protocol article provides a detailed experimental protocol to the method presented in the experimental article (E.V. Kozar, E.G. Kozar, E.A. Domblides. Effect of the Method of Microspore Isolation on the Efficiency of Isolated Microspore Culture In Vitro for Brassicaceae Family. Horticulturae. 2022. Vol. 8, No. 10. P. 864. DOI 10.3390/horticulturae8100864) but does not repeat all the results documenting the efficacy of the actual method.

## Introduction

The development and implementation of cell technologies have changed the breeding process of agricultural plants in the world. Doubled haploids (DH)-technologies significantly accelerate the creation of hybrids by producing pure lines (potential parental lines) in 1–2 years, while traditional breeding produces such lines in 6-12 years depending on the crops [1].

DH are obtained in anther culture, in microspore culture, and in unfertilized ovule culture. Microspore culture is prioritized because only in this culture there are no somatic tissues, thus avoiding the additional step of testing plants for their origin from haploid cells. The method for obtaining DH in microspore culture is well established for a number of crops. However, even among responsive crops (rapeseed, white cabbage) not all genotypes are equally responsive to this method, so it is constantly being improved [2–4].

The protocol for obtaining DH *in vitro* microspore cultivation consists of a large number of steps, each of which has a great impact on the final result [5–9]. The general technological scheme can be divided into two major stages: (i) induction of embryogenesis in isolated microspore culture *in vitro*; (ii) the stage of regeneration of regenerant plants from embryos. The first stage includes: cytological analysis of buds; selection of buds of optimal size; sterilization of buds; isolation of microspores from buds; cultivation of microspores on liquid nutrient medium until embryos at the cotyledonary stage of development are obtained. The second stage includes: regeneration of embryos on solid nutrient medium with induction of shoots *in vitro*; cultivation of microshoots on solid nutrient medium with induction of rhizogenesis *in vitro*; rooting and adaptation of regenerated plants under *ex vitro* conditions. There are different modifications for all the above steps such as different heat stress composition of nutrient media, ways of planting microshoots on nutrient media, etc. [8, 10]. The protocol described below incorporates the best modifications of each step according to our personal experience and literature data. The main difference between this protocol and all previously published protocols is the modified microspore isolation step (steps 11–12 in section Culture of microspore in vitro) that we developed in our laboratory [11].

In standard protocols for obtaining DH *in vitro* microspore culture, microspores are isolated by destroying buds immersed in nutrient medium: in test tube with magnets on a magnetic stirrer [2, 12]; by crushing buds by rotary movements in test tubes with a piston (syringe) [4]; by crushing buds with a pestle in a mortar [13]; by crushing buds with a glass rod in a test tube [14] or by crushing buds in a blender [15].

We propose a new method for microspore isolation, which consists of individually a transverse cutting buds with scalpel, then immersing the bud halves in sterile tubes with nutrient medium and shaking them on a rotary shaker for 10–60 s, depending on the genotype and crops (selected empirically).

## Materials

### Reagents

1. DAPI/Antifading solution, ready to use (Liquid PBS pH 8.0 containing ∼50% glycerol and 0.4 μg/ml of DAPI and 2–7mM phenylenediamine)
2. 96% Ethanol
3. NaOH and HCl
4. NaOCl
5. Autoclaved distilled water (ADW).
6. Liquid fertilizer (N—13%, P_2_O_5_—5%, K_2_O—25%, MgO—2%, S—8%, Fe (EDTA) 0.054%, Zn (EDTA)—0.014%, Cu (EDTA)—0.01%, Mn (EDTA)—0.042%, Mo—0.004%, B—0.02%).
7. NLN medium [16]—Basal Salt (without vitamins, without calcium nitrate and sucrose)
8. NLN medium vitamin mixture
9. Murashige and Skoog medium (MS) [17]—Basal Medium (with vitamins, without sucrose and agar)
10. Ca(NO_3_)_2_·4H_2_O
11. Activated carbon
12. Agarose
13. Gibberellic acid (GA)
14. 6-benzylaminopurine (BA)
15. Tween 20
16. Agar for plant tissue culture
17. Sucrose for plant tissue culture
18. Ultrapure water type 1 quality (UDW)
19. Propidium iodide (PI)
20. RNase A (10 mg/ml)
21. MgCl_2_
22. MOPS
23. Sodium citrate
24. Triton X-100

### Recipes

1. Substrate mix: peat, vermiculite, sand in the proportions 6:1:1.
2. Liquid medium NLN-13 [16]: Dissolve 0.392 g medium NLN Basal Salt, 1.04 g NLN medium vitamin mixture, NLN 0.5 g Ca(NO_3_)2·4H_2_O and 130 g sucrose in UDW and bring the volume to 1 L. Adjust the pH to 5.8 with 1 M NaOH. Sterilize the medium using a vacuum pump and a PES filter system with a pore diameter of 0.22 µm in a laminar flow cabinet (shelf life 1 month 4°C).
3. Solid medium MS [17]: Dissolve 4.4 g MS medium with vitamins and 20 g sucrose in UDW and bring the volume to 1 L. Adjust the pH to 5.8 with 1 M NaOH. Prepare two 1 L bottles for sterilizing the medium. Add 3.5 g agar and 500 ml of prepared medium to each 1-L bottle. Autoclave for 25 min at 121°C, 1.1 kPa. Transfer to a laminar flow cabinet. Wait for the medium to cool down to 50–60°C. Mix the medium and pour it into sterile culture vessels (the thickness of the medium in the culture vessel should be 1–2 cm) (shelf life 1 month 25°C).
4. Activated carbon with agarose (AC): Dissolve 1 g of activated carbon and 0.5 g of agarose in UDW in a 100-ml glass bottle and bring the volume to 100 ml. Autoclave the bottle with AC; store in a laminar flow cabinet under sterile conditions (shelf life 3 months at room temperature).
5. GA and BA stock solutions: Dissolve GA in 96% ethanol to a concentration of 1 mg/ml. Dissolve BA in a small amount of 1 N NaOH and adjust with ADW to a concentration of 1 mg/ml. Sterilize solutions using sterile syringes and sterile syringe PES membrane filters 0.2 μm. Dispense 1 ml aliquots in Eppendorf-type microtubes (shelf life 3 months 4°C).
6. Hormonal solid medium MS: Prepare solid medium MS as described in section Recipes 3 with the following modifications—after cooling the medium to 50–60°C, add 50 µl GA and 500 µl BA to each 1 L bottle containing 500 ml of medium. Mix the medium and pour into sterile culture vessels (the thickness of the medium in the culture vessel should be 1–2 cm).
7. Disinfecting solution: 4°C ADW, 5% NaOCl in a 1: 1 ratio and 2 drops of Tween.
8. Galbraith buffer [18]: Dissolve 0.045 g MgCl_2_, 0.418 g MOPS, 0.774 g sodium citrate, 100 μl Triton X-100 in UDW and bring the volume to 100 ml. Adjust the pH to 7.0 with 1 M NaOH (storage time 1 month at 4°C).
9. PI stock solution: dilute powder in UDW to stock concentration 1/ml. Take time to fully dissolve PI. CAUTION: MUTAGEN, TOXIC. Handle with gloves, do not inhale (store in a dark location, shelf life 3 months 4°C).

### Equipment

1. Growth room with controlled photoperiod (16/8 h, light intensity of 65 μmol m^−2^s^−1^) and temperature (constant l9°C)
2. Lighting installation with controlled photoperiod (16/8 h, light intensity of 20 μmol m^−2^s^−1^) and air conditioning (23°C)
3. Autoclave
4. Balance
5. pH meter
6. Magnetic stirrer
7. Rotary shaker
8. Burner
9. Fridge and freezer
10. Distiller and ultrapure water purification system
11. Refrigerated centrifuge
12. Laminar flow cabinet
13. Incubators with controlled temperature (25 and 32°C)
14. Vacuum pump for sterilizing solutions through filters
15. Microwave
16. Light/UV microscope with filter combinations for DAPI
17. Stereomicroscope
18. Inverted microscope
19. Flow cytometer with the fluorochrome excitation wavelength—535 nm for PI (e.g., CytoFLEX Beckman Coulter, USA).

### Tools

1. Plastic pots (volume 0.3 L, 0.5 L, 1 L, 5 L, 10 L)
2. Slides and coverslips
3. Dissecting needles
4. 250 ml jars
5. 100 ml glass bottles
6. Nylon filters (pore size 40μm) for Falcon-type tubes
7. 7 × 7 cm pieces of fabrics (the cloth should be well permeable to liquid) with thread
8. 1.5 ml Eppendorf type microtubes
9. Syringes 10 ml
10. 100- and 300-ml culture vessels
11. Glass 90-mm diameter Petri dishes
12. Tweezers
13. Scalpels
14. Racks for 50 ml tubes and 1.5 ml microtubes
15. 5–50 μl and 500–1000 μl variable volume pipettes
16. 30 µm pre-separation filter
17. 5 ml sterile Pasteur pipettes
18. 50 ml sterile Falcon-type tubes with screw cover
19. 6-cm diameter sterile Petri dishes
20. Parafilm or equivalent sealing film
21. Cotton wool
22. Sterile syringe PES membrane filters 0.2 μm
23. Bottle-top vacuum polyethersulfone (PES) filter system with pore diameter 0.22 μm.
24. Blades
25. Cylindrical tube with screw cap sterile 10 ml

## Procedure

### Donor plants and growth conditions

1. Seeds are sown in 0.3 L plastic pots filled with a mix of substrate (section Recipes 1).
2. Place pots with seeds in the growth room under a 16-h photoperiod with light intensity of 65 μmol m^−2^s^−1^ at constant l9°C.
3. When the plants are at the 2–3 leaf stage, start watering three times a week with 0.1 g/L liquid fertilizer (section Reagents 6), before that water the plants without fertilizer.
4. While the plants are growing, transfer them to larger plastic pots (0.5–10 L, repeat as needed). Do not transfer plants if they start to blossom.
5. Use the inflorescences as donors for microspore culture after the first 1–3 flowers are open.
6. Remove open flowers throughout flowering to prevent pod development.

### Developmental stage of the microspores

**Figure 1.**
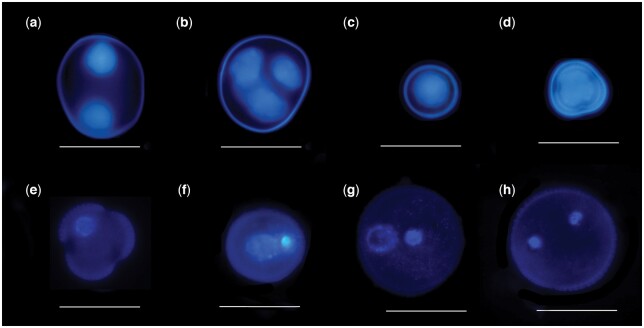
DAPI staining of microspores. (**a**) Dyad stage, (**b**) tetrad stage, (**c**) early unicellular stage, (**d**) mid unicellular stage, (**e**) late vacuolated unicellular stage, (**f**) early pollen stage, (**g**) mid pollen stage, (**h**) mature pollen stage. Bars: 20 μm.

1. Measure the length of the bud with a stereomicroscope.
2. Apply a drop of DAPI/Antifading solution on the glass slide and remove anthers from buds and squeeze the anthers to release the microspores into the drop using dissecting needles.
3. Dissecting needles are then used to separate the microspores from the anther parts.
4. Anthers are removed from the drop and the drop with microspores is covered with a coverslip.
5. The glass slide with the microspores sample is left in the dark for 1 h.
6. After that, the samples can be observed under a UV microscope and the stage of development of microspores can be studied.
7. Examine the microspore sample (Fig. 1).
8. Assess the stage of development of microspores of several different sizes of buds.
9. Select the sample with the highest concentration of microspores in the late vacuolated unicellular and early bicellular stages and remember this bud size (Fig. 1 e, f).
10. Use this bud size to cultivate microspores with a range of ±0.25 mm relative to this size.

### Culture of microspore *in vitro*

**Figure 2.**
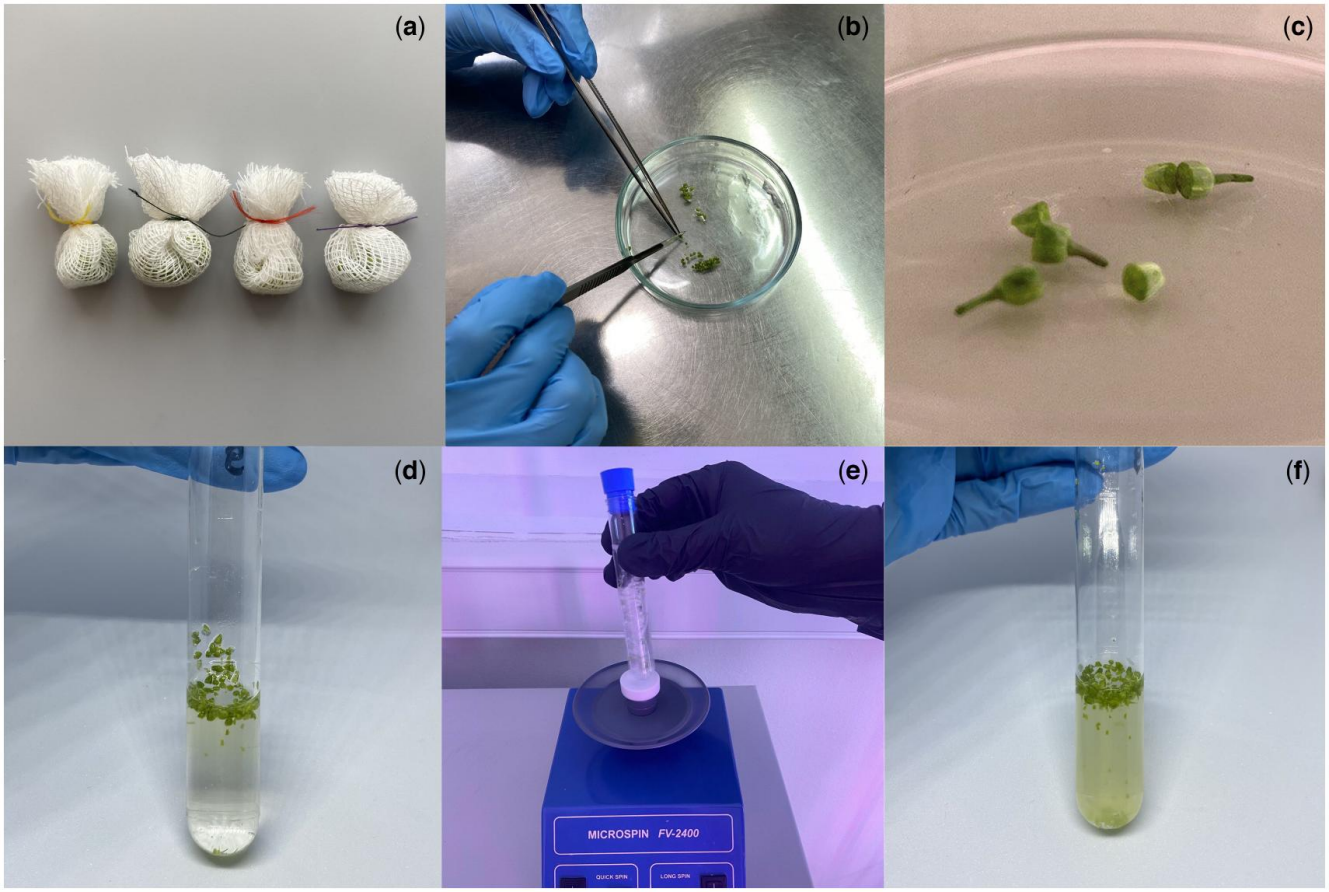
(**a**) Bags with buds, (**b**) bud cutting, (**c**) halved buds, (**d**) sterile 10 ml tubes filled with medium and buds, (**e**) shaking the tube on a rotary shaker, (**f**) test tubes with microspores and buds after shaking.

**Figure 3.**
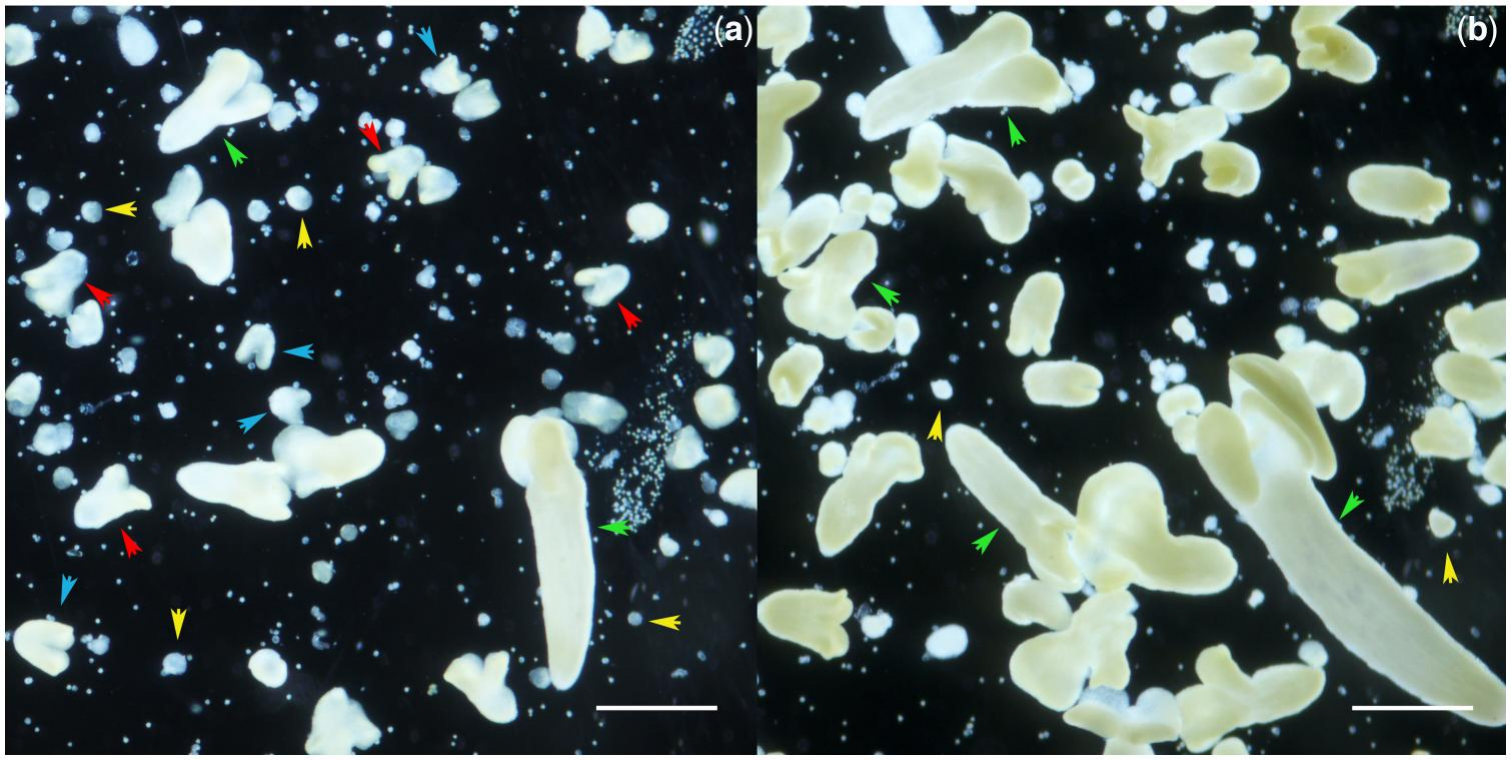
View of petri dishes during cultivation of rape microspores. (**a**) day 17 of cultivation, (**b**) day 30 of cultivation. Yellow arrows—globular stage of embryo development, blue arrows—heart-shaped stage of embryo development, red arrows—torpedo-shaped stage of embryo development, green arrows—cotyledonary stage of embryo development. Scale bar 2000 μm.

1. All tools used must be sterile. If they are not single-use sterile tools, they must be autoclaved before use. Forceps and scalpels are sterilized with 96% ethanol flame sterilized during operation in a laminar flow cabinet before each new manipulation. All media used in the procedure must be cooled to 4°C. To do this, store liquids in the refrigerator and take them out just before use.
2. Inflorescences are removed from donor plants and placed in containers with moist absorbent cotton. They are then placed in the refrigerator (4°C) for 24 h.
3. Before starting bud sampling, disinfect the laminar flow cabinet with 96% ethanol and turn on the UV mode for 15 min. Then turn off the UV mode and turn on the normal light and laminar flow.
4. Turn on the refrigerated centrifuge.
5. The inflorescences are then removed from the refrigerator and the buds are selected using tweezers and a stereomicroscope according to the length determined in section Developmental stage of the microspores 9–10.
6. Count the selected buds, wrap them in pieces of cloth (section Tools 7) and secure them with thread (bud bags) (Fig. 2 a). Place any number of buds in each bag, but make sure that the buds in the bags are not pressed tightly together so that the sterilizing solution can penetrate between all the buds.
7. All subsequent operations are performed in a laminar flow cabinet under sterile conditions. Disinfect hands with 70% ethanol before starting work.
8. Place the magnet and bud bags in a 250 ml jar of chilled (4°C) 96% ethanol. Then place the jar on a magnetic stirrer and stir for 30 s.
9. Transfer the magnet and bud bags with sterile tweezers to a 250 ml jar with disinfecting solution at 4°C (section Recipes 7), then place the jar on a magnetic stirrer. Stir for 15 minutes.
10. Transfer the magnet and bud bags with sterile tweezers into a 250-ml jar with ADW (4°C), then place the jar on a magnetic stirrer. Rinse for 10 min. Repeat three times.
11. Transfer the bud bags with sterile tweezers to a sterile glass Petri dish (section Tools 11), cut the threads with a sterile scalpel to pull out the buds. Each bud should be cut across into two halves using a sterile scalpel. To do this, the bud must be held with sterile tweezers, as the incision must be made without pressing on the bud (Fig. 2 b, c). Use new blades so that the bud tissue cannot be crushed while cutting. Transfer the bud halves carefully into sterile 10-ml screw-capped tubes with liquid nutrient medium NLN-13.
12. Place the screw-capped tubes with the buds on a rotary shaker for 10–60 s (Depending on the crops. The more fragile the buds are, the less time you need to shake them. Do not shake too vigorously or the effect of the new type of isolation will be lost). Shaking the tubes on a rotary shaker should cause the microspores to spill out into the nutrient medium.
13. Prepare a rack with sterile 50 ml Falcon tubes. Open the tubes and place a sterile nylon filter (40 µm pore diameter). The number of tubes should match the number of samples and be even numbered, as these tubes will be placed in the refrigerated centrifuge.
14. The contents of 10-ml screw cap tubes are transferred to a nylon filter using a sterile Pasteur pipette to filter into Falcon tubes and brought to 15 ml volume using chilled (4°C) medium NLN-13. Then close the lids, place the tubes in a refrigerated centrifuge and spin for 5 min at 100–130 g.
15. After centrifugation, carefully remove the supernatant using a sterile Pasteur pipette. Then, using a new Pasteur pipette, bring the volume in the Falcon tubes to 15 ml using chilled (4°C) medium NLN-13. Then close the lid and mix the precipitate. Place the tubes in a refrigerated centrifuge for 5 min at 100–130 g. This operation should be repeated twice.
16. During the last centrifugation, prepare Petri dishes (section Tools 19) and melt the AC in a microwave oven. Drop 3-4 drops of AC (section Recipes 4) into each 6 mm sterile Petri dish. Wait for the AC to solidify. Then add 4 ml of medium NLN-13 (4°C) (section Recipes 2) to each sterile Petri dish.
17. After the last centrifugation and supernatant removal, add chilled (4°C) medium NLN-13 to the tubes at a rate of 1 ml of medium per 5 buds. Mix the suspension well with a sterile Pasteur pipette and pour 1 ml of the suspension into each sterile Petri dish (each Petri dish should end up with 5 ml of medium NLN-13 and a suspension of microspores from 5 buds, corresponding to an approximate microspore density of 100,000 microspores per 1 ml, you can count microspores with a hemacytometer for greater accuracy).
18. Seal the sterile Petri dishes with Parafilm M elastic band or food film, sign them and place them in an incubator for 24 h at 32°C.
19. After 24 h, the Petri dishes are transferred to an incubator at 25°C until microspore embryos (MDEs) appear at the cotyledonary stage of development (21–30 days). Observe the formation of MDEs using an inverted microscope throughout the cultivation period.
20. The obtained embryos at the cotyledonary stage were transferred to a light room with controlled photoperiod (16/8 h, illumination 2500 lux) and air conditioning (23°C) for 48 h and then transferred to solid nutrient medium (Fig. 3). During this time, the embryos turn green.

### Regeneration stage

1. Under sterile conditions, transfer the MDE with sterile tweezers into sterile cultivation vessels containing solid MS medium with 0.1 mg/L GA and 1 mg/L BA for 7–20 days. The MDE is placed strictly on the surface of the solid medium without going deep into it.
2. After formation of secondary MDEs, transfer the structures with secondary embryos using sterile tweezers into sterile glass petri dishes. Separate secondary embryos with a sterile scalpel from other structures (callus). Transfer secondary embryos with sterile forceps into individual culture vessels with solid hormone-free MS medium.
3. Embryo tissues differentiate during the cultivation process. The growing point is formed, as well as the first leaves and roots. As regenerated plants form, transfer plant explants into a fresh sterile culture vessel with hormone-free MS solid medium at least once a month.
4. After the formation of root system and 3-4 leaves, transfer the regenerated plants to *ex vitro* conditions.

### Adaptation of regenerant plants to the soil

1. Substrate (section Recipes 1) for rooting of regenerated plants should be sterilized in a dry oven at 100°C for 1 h. in a 101-iron container filled not more than half full.
2. Regenerated plants with a formed root system are removed from the culture vessels and thoroughly washed under a stream of water at room temperature to remove agar residues.
3. Plants are transferred into plastic pots of 0.3 L with a substrate.
4. The transferred plants are covered with clear plastic cups and placed in the growth room under a 16-h photoperiod with light intensity of 65 μmol m^−2^s^−1^ at constant l9°C.
5. Feeding with 0.1 g/L complex fertilizer (section Reagents 6) is carried out three times a week starting from the second week after transferring.
6. Plastic cups are removed when regenerated plants have 1–2 new leaves that were formed after transferring.
7. Further transfers into larger pots are carried out in a standard way as the plants grow.

### Ploidy estimation of plants [19]

1. Pick 1–2 young leaves (1–2 cm^2^) from the reference plants, as well as from plants of unknown ploidy. The plants of the same species/cultivar with known ploidy can be used as external standards.
2. Add 50 µg/ml of RNase A to Galbraith buffer immediately before use.
3. Slice leaves of control plants with a razor blade in a petri dish with 500 μl of Galbraith ice-cold buffer [18], supplemented by RNase A.
4. Filter the sample through a 30 µm pre-separation filter and transfer to a sample tube.
5. Add propidium iodide (PI) to a final concentration of 50 µg/ml. The optimal PI staining time should be determined for each species separately.
6. Adjust the voltage of the photomultiplier/photodiode (Gain) of the flow cytometer and set up gating. Further analysis of all samples is performed without changing the instrument settings.
7. Register the PI-stained nuclei. Measure the mean of the G1/G0 peak on the PI histogram. Perform three measurements of reference leaf nuclei and use the average value for further calculations with at least 1000 (preferably more than 5000) counts.
8. Analyze the plants with unknown ploidy using the same sample preparation procedure steps 3–5.
9. Record the test sample peak mean value with at least 5000 counts.
10. Determine ploidy using the formula: Sample ploidy = Reference ploidy × Sample mean of the G0/G1 peak/Reference mean of the G0/G1 peak.
11. Process the data from three independent experiments using statistics software.
12. Overlayed reference and sample PI histograms can be used for visual data representation.

## Discussion

Standard types of microspore isolation are based on complete mechanical disruption of bud and anther tissues followed by filtration. This isolation of microspores is suitable for cultivations of the Brassicaceae family, which are very responsive to embryogenesis, suggesting a low concentration of dead microspores in the cultivation and therefore a low concentration of toxins in the cultivation compared to cultivations where a significant proportion of microspores die during cultivation process.

On the other hand, with high responsiveness of microspores to technology, the sensitivity of embryos yield to different modifications of technology is not significant. Therefore, for crops that are well responsive to embryogenesis, the method of microspore isolation does not play a significant role, whereas for poorly responsive crops, the method of isolation has a significant impact.

The new protocol, thanks to a modified method of microspore isolation, allows to significantly increase the efficiency of embryo production in microspore cultivation (up to 7.5 times for example for mustard) for poorly responsive crops, and sometimes to obtain the first embryos (e.g. for red cabbage) [11].

Thus, if you encounter poorly responsive crops and/or genotypes we recommend trying out this protocol.

Note that this protocol focused on the method of microspore isolation, so one possible option is considered for the other factors. The steps that can vary depending on the genotype are duration of cold treatment of buds before introduction into culture, composition of nutrient media for induction of embryogenesis, density of microspores in the preparation, duration of temperature stress at 32 degrees, composition of nutrient media for in vitro regeneration and rooting, and others. Also, all dishes and technical devices used in the protocol can be exchanged for other ones if such functionality is preserved.

## Author contributions

Elena Victorovna Kozar (Conceptualization [equal], Data curation [equal], Formal analysis [equal], Investigation [equal], Methodology [equal], Visualization [equal], Writing—original draft [equal]) and Elena Alekseevna Domblides (Project administration [supporting], Supervision [supporting], Validation-Supporting, Writing—review & editing [supporting])

*Conflict of interest statement*. None declared.

## Funding

None declared.
